# Advances in the study of the role of high-frequency mutant subunits of the SWI/SNF complex in tumors

**DOI:** 10.3389/fonc.2024.1463892

**Published:** 2024-12-04

**Authors:** Jiumei Zhao, Jing Zhu, Yu Tang, Kepu Zheng, Ziwei Li

**Affiliations:** ^1^ Chongqing Nanchuan District People’s Hospital, Chongqing, China; ^2^ Kunming Medical University, Kunming, China; ^3^ The Third Affiliated Hospital of Kunming Medical University, Kunming, China; ^4^ Chongqing Health Center for Women and Children, Women and Children’s Hospital of Chongqing Medical University, Chongqing, China

**Keywords:** SWI/SNF complex, ARID1A, SMARCA4, tumor, tumor resistance, molecular mechanism, targeted therapy

## Abstract

SWI/SNF (Switch/Sucrose non-fermentable, switch/sucrose non-fermentable) chromatin remodeling complex is a macromolecular complex composed of multiple subunits. It can use the energy generated by the hydrolysis of ATP (Adenosine triphosphate) to destroy the connection between DNA and histones, achieve the breakdown of nucleosomes, and regulate gene expression. SWI/SNF complex is essential for cell proliferation and differentiation, and the abnormal function of its subunits is closely related to tumorigenesis. Among them, ARID1A, an essential non-catalytic subunit of the SWI/SNF complex, can regulate the targeting of the complex through DNA or protein interactions. Moreover, the abnormal function of ARID1A significantly reduces the targeting of SWI/SNF complex to genes and participates in critical intracellular activities such as gene transcription and DNA synthesis. As a catalytic subunit of the SWI/SNF complex, SMARCA4 has ATPase activity that catalyzes the hydrolysis of ATP to produce energy and power the chromatin remodeling complex, which is critical to the function of the SWI/SNF complex. The study data indicate that approximately 25% of cancers have one or more SWI/SNF subunit genetic abnormalities, and at least nine different SWI/SNF subunits have been identified as having repeated mutations multiple times in various cancers, suggesting that mutations affecting SWI/SNF subunits may introduce vulnerabilities to these cancers. Here, we review the mechanism of action of ARID1A and SMARCA4, the two subunits with the highest mutation frequency in the SWI/SNF complex, and the research progress of their targeted therapy in tumors to provide a new direction for precise targeted therapy of clinical tumors.

## Introduction

The annual global incidence of cancer is still on the rise, and cancer remains a significant health challenge worldwide (1). According to the World Health Organization (WHO), there will be 19,976,499 new cases of malignant tumors globally in 2022, an increase of nearly 700,000 from 2020. There will be 9,743,832 new deaths from malignant tumors globally in 2022 (2). Currently, cancer patients are treated with surgical resection, radiotherapy, chemotherapy, small molecule targeted drug therapy, etc. For patients who are not suitable for surgery and radiotherapy, the modalities of chemotherapy and targeted drug therapy are essential, and patients treated with chemotherapy and targeted drug therapy usually develop secondary resistance after 6-12 months, which ultimately leads to cancer deterioration (3–5). Therefore, an in-depth investigation of the cancer development process can help to improve the survival of cancer patients.

Cancer development and progression is a multigene, multistep, and multistage process in which the disorganization of chromatin organization is an essential feature in the development of cancer (6, 7). In the eukaryotic nucleus, the functional unit of chromatin is made up of DNA and histone octamers, which are assembled from 2 molecules, H2A, H2B, H3, and H4, where histone H1 attaches to the DNA outside of the core particles, thereby locking the inner and outer ends of the nucleosome DNA and stabilizing the nucleosome (8, 9). Epigenetic modifications play an essential role in tumor development, including DNA methylation, histone modifications, non-coding RNA modifications, and chromatin remodeling, which regulate essential cell physiological processes and maintain cellular properties. Chromatin topology can be altered by proteins that modify DNA itself, modifying proteins of histones, and ATP-dependent chromatin remodeling complexes to regulate gene expression (8, 9). DNA modifying proteins regulate the transcriptional activity of the corresponding genes by covalently modifying DNA through cytosine methylation. On the other hand, histone modifications mediate covalent post-translational modifications of histone globular structural domains or histone tails, thereby altering chromatin compression, nucleosome dynamics, or recruitment of other chromatin-binding proteins (8, 9). ATP-dependent chromatin remodeling complexes alter chromatin accessibility, regulate gene expression, and influence cell division and differentiation as well as intracellular homeostasis by harnessing the energy generated by ATP hydrolysis to reposition, eject, slide, or alter histones, which are the constituent components of nucleosomes (8–10).

The ATP-dependent chromatin remodeling complexes families (CRC families) contain four members: SWI/SNF (switch/sucrose non-fermentable), imitation SWI (imitation switch, ISWI), INO80 (INOsitol requiring 80), and CHD (chromodomain helicase DNA-binding), all of which are macromolecular complexes consisting of many different variable subunits that can affect nucleosome composition, make DNA binding and easier recruitment of proteins and transcription elements. Proteins and transcription elements are more likely to recruit DNA for gene expression (10).

Within the CRC family, mutations in the subunits encoding the SWI/SNF complex are the most common. The SWI/SNF complex was first identified in Saccharomyces cerevisiae (Saccharomyces cerevisiae) for both mating switching (the SWI phenotype) and sucrose fermentation (the sucrose non-fermentation phenotype), hence its name (11). From yeast to humans, the SWI/SNF complex has generally remained highly conserved in its evolution, although it has continuously changed its subunit composition during evolution in order to adapt to the gradually increasing genome complexity (11). The SWI/SNF complex is a crucial regulator of nucleosome localization and has been implicated in controlling specific transcriptional programs, such as those mediating cellular differentiation and lineage-specific transcriptional programs. In addition, it is involved in regulating various critical cellular processes, such as cell cycle, cell morphology, adhesion, apoptosis, signal transduction, DNA damage repair, and stress response, among other biological processes (12, 13).

**Figure 1:**
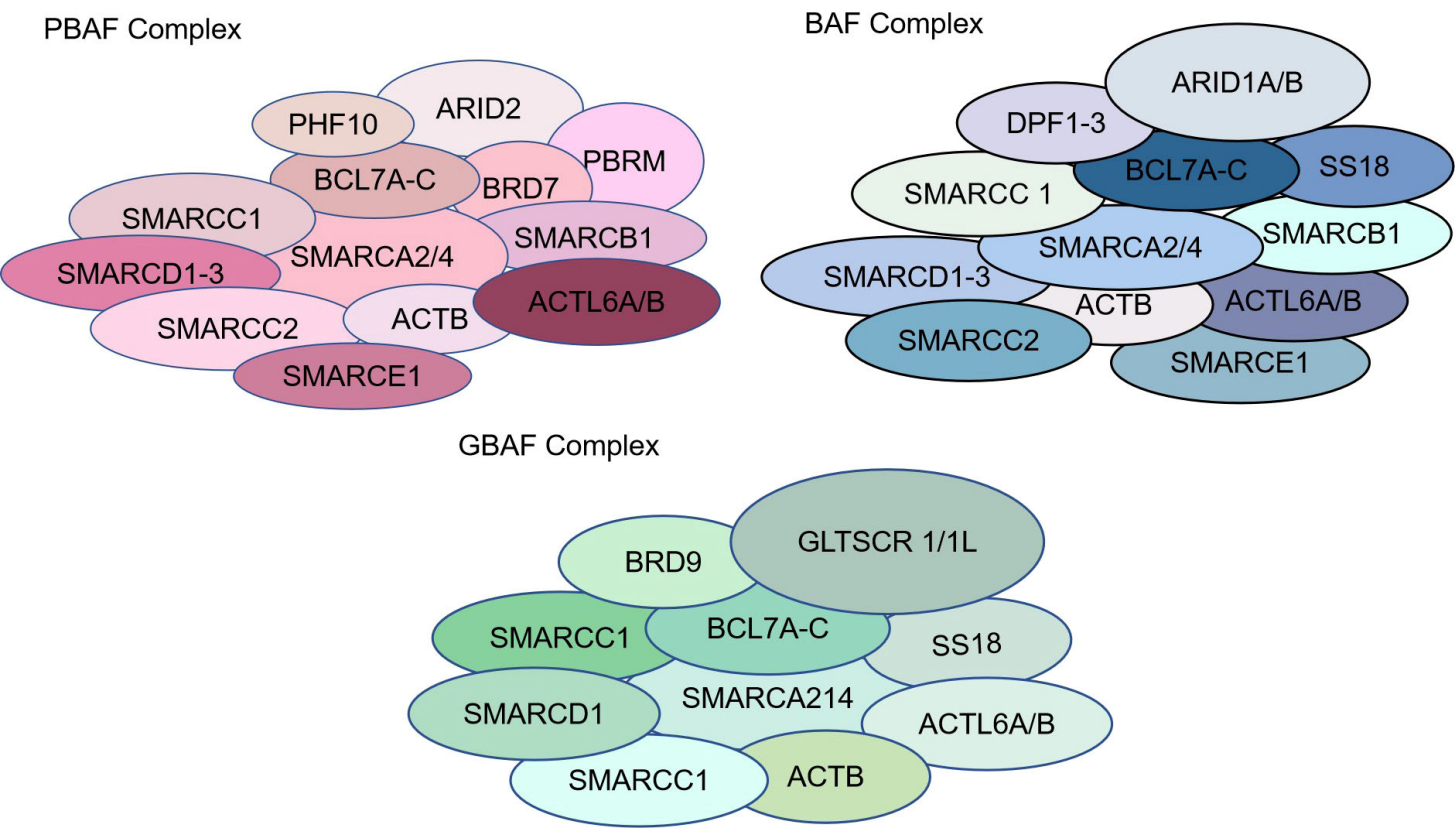
Types and structures of SWI/SNF chromatin remodeling complexes. The SWI/SNF complex contains three isoforms: classical BAF (canonical BRM/BRG1-associated factor, cBAF), polybromine-associated BAF (PBAF), and atypical BAF (non-canonical BAF, ncBAF).

The SWI/SNF complex contains three isoforms: classical BAF (canonical BRM/BRG1-associated factor, cBAF), polybromine-associated BAF (PBAF), and atypical BAF (non-canonical BAF, ncBAF) (**Figure 1**) (14). All three SWI/SNF complex isoforms contain the core subunits SMARCC1, SMARCC2, SMARCD1 and the ATPases SMARCA4 (BRG1) and SMARCA2 (BRM), with SMARCC1, SMARCC2, and SMARCD1 comprising the initial BAF core, which is an intermediate in the SWI/SNF complex assembly process (14). In addition, core subunits of cBAF and PBAF include SMARCB1 and SMARCE1. SMARCC1 and SMARCC2 are homologous or heterodimers, there are regions on the SMARCC subunit that bind to SMARCE1, SMARCD, and ARID1 subunits, and when the SMARCC subunit is missing, the entire chromatin remodeling complex is actually degraded. Loss of SMARCD subunits inhibits the binding of ARID, SMARCA4, and SMARCA2 to the core complex, loss of SMARCE1 reduces complex stability, and loss of SMARCB1 results in partial breakdown of the complex (15–17). Subunits unique to cBAF include ARID1A/B and DPF1/2/3; subunits unique to PBAF include ARID2, PBRM1, PHF10, and BRD7, and GLTSCR1, GLTSCR1L, and BRD9 are subunits unique to ncBAF (15, 16). The SWI/SNF complex consists of an ATPase, an actin-related protein (The ATPase module contains Snf2 (SMARCA4/SMARCA2), the ATPase of Snf2 consists of a pair of bracketed helices joined by two RecA-like domains, which form a cleft with the nucleosomal DNA exposed at the superhelical location 2 (SHL2) of the nucleosome. Location 2 (SHL2); Snf2 also includes a C-terminal bromodomain, which mainly serves to increase the affinity for acetylated nucleosomes, while the ARP module consists of the Snf2 HSA (helicase-SANT associated) domain, Rtt102, Arp7, and Arp9, which are responsible for the support of the coordinating ATPase and the main module. The ARID1 subunit, on the other hand, exists as a core binding region consisting of an N-terminal, AT-rich interaction domain (ARID) and three C-terminal potential structural domains. The ARID structural domains are mainly responsible for cross-linking the complex to DNA without participating in the assembly of the complex. SWI/SNF complexes are mainly enriched in enhancer regions and act at promoters, gene bodies, and other sites involved in the activity of transcription factors and other chromatin regulators in the regulation of gene expression (18). The SWI/SNF complex is also involved in DNA damage repair (DDR), and there is evidence that the SWI/SNF complex contributes to successful repair through homologous recombination and non-homologous end-joining pathways. For example, ARID1A plays an epigenetic role in promoting the DNA double-strand breaks (DSBs) repair pathway, non-homologous end joining (NHEJ), and homologous recombination (HR). Whereas SMARCA2/4 can migrate to DNA lesions during DNA damage to participate in DNA damage repair, lack of SMARCA2/4 causes γH2AX, ring finger protein 8 (RNF8), and breast cancer susceptibility gene 1 (BRCA1) to linger at DNA lesions for just a more extended period and impairs RAD51-dependent homologous recombination repair (19–21).

Mutations in the SWI/SNF complex are found in approximately 25% of all human cancers. There are two types of mutations in the SWI/SNF complex, one is inactivating mutations, to which most subunit mutations belong. The other is gain-of-function mutations, which are SS18-SSX fusion mutations that result in higher nucleosome mobilization activity in the SWI/SNF complex. The types of mutations in the genes encoding the SWI/SNF subunits of the SWI/SNF complex are nonsense mutations, shifted code mutations, missense mutations, and deletion mutations (12, 22).

In summary, the SWI/SNF complex is involved in developing a wide range of cancers, and the different subunits appear to have a bias towards different cancer types rather than a random distribution. When a subunit in the SWI/SNF complex is mutated, the interactions between the subunits within the complex are altered, leading to disruption of the normal assembly pathways of the subunits of the complex and stabilization of the subunit proteins, which may also lead to altered accessibility of chromatin enhancers and promoters, resulting in the transcriptional activity of genes being compromised, and thus causing cancer. In this paper, we will focus on the role of the two most frequently mutated subunits of the SWI/SNF complex, ARID1A and SMARCA4, in tumors and their molecular mechanisms, as well as the anti-tumor therapeutic strategies that have been identified for the corresponding mutations. It aims to provide new ideas for basic research on SWI/SNF-related subunit mutations and to provide a reference for developing tumor-targeting drugs induced by their related mutations.

## ARID1A alterations and tumors

The protein BAF250A encoded by the ARID1A gene is one of the essential subunits of the SWI/SNF chromatin remodeling complex, and in a wide range of tumors, ARID1A is the most commonly mutated subunit in the SWI/SNF complex and one of the most commonly mutated oncogenes in human cancers (23). Mutations in the ARID1A gene mainly lead to its inactivation and absence of ARID1A protein expression. Several studies have shown that inactivation of ARID1A can affect the biological functions of tumor cells, such as proliferation, metastasis, differentiation, apoptosis, and drug sensitivity, and is closely related to the clinical prognosis of tumors (24). Mutations in ARID1A have been found in a variety of tumors (25). In 2010, researchers collected cancerous and normal tissue samples from eight ovarian cancer patients and identified and first reported ARID1A gene mutations by exome sequencing and Sanger sequencing in 57% of patients with clear cell carcinoma of the ovary. In addition, the mutations in ARID1A all occurred in the coding region, and these mutations led to the appearance of stop codons through base substitutions, resulting in the translation of ARID1A to form a truncated protein (26). Then, successive studies have shown that ARID1A mutations are also present in other cancers, such as 29% in gastric cancer, 10-17% in hepatocellular carcinoma, 10% in colorectal cancer, and 13% in bladder cancer (23, 27–31). In conclusion, the mutation rate of ARID1A in all cancers is about 8% (32).

## ARID1A acts primarily as a tumor suppressor

### ARID1A is involved in the regulation of cell proliferation, migration, and apoptosis

Studies have shown that ARID1A is involved in cell proliferation, migration, and apoptosis and plays a significant role in tumor inhibition (23).ARID1A expression is correlated with cell cycle changes, and the expression of ARID1A is highest in the G0-G1 phase, significantly reduced in the S and G2-M phases, and almost entirely absent in actively dividing cells (33). It was found that ARID1A could act directly or indirectly to regulate p21/WAF1 gene expression and thus affect cell proliferation. Mechanistically, p21/WAF binds to and inhibits the activity of the cell cycle protein CDK2/CDK4 complex, thereby contributing to cell arrest in the G1 phase (25). In addition, long-chain non-coding RNAs (lncRNAs) DGCR5 interacts with ARID1A, promoting the transcription of p21 and thus affecting cell proliferation (34). In addition, it was found that down-regulation of the ARID1A gene in gastric and breast cancer cells significantly enhanced cell proliferation. In contrast, the proliferation of cells replying to ARID1A gene expression was inhibited (35). In addition, ARID1A is closely related to cell invasion. It has been shown that the activation of epithelial-mesenchymal transition (EMT) and stem cell recognition pathways partially depends on the deletion of ARID1A. Knockdown of ARID1A enhanced migration and invasion of gastric cancer cells, and exogenous overexpression of ARID1A inhibited cell migration. In addition, the knockdown of ARID1A and cell adhesion protein (E-cadherin) were down-regulated, and the overexpression of ARID1A resulted in the up-regulation of E-cadherin expression (36). This suggests that low expression or deletion of ARID1A enhances migration and invasion of gastric cancer cells by down-regulating E-cadherin. Furthermore, the knockdown of ARID1A in hepatocellular carcinoma and breast cancer cells significantly enhanced cell invasion and migration. ARID1A was also found to play a tumor suppressor role in nasopharyngeal carcinoma (NPC), and deletion of ARID1A activated the AKT signaling pathway to promote tumor cell migration and invasion (37). ARID1A was negatively correlated with the expression of MCL-1, an anti-apoptotic protein in colorectal cancer (38), and ARID1A could inhibit tumor growth by down-regulating MCL-1 (39). Knockdown of ARID1A in non-small cell lung cancer (NSCLC) resulted in up-regulation of the expression of cycle-related proteins cyclinD1 and Bcl-2 and inhibition of apoptosis (34). The ARID1A gene may exert tumor suppressive effects by affecting the above three cellular processes.

### ARID1A regulates DNA repair to exert its oncogenic effects

Changes in the external biochemical environment and cellular metabolites can lead to DNA damage, leading to aberrant DNA replication and transcription processes, and inactivation of DNA damage repair pathways under replication stress will lead to cell death or cancer (40, 41). Chromatin remodeling complexes are crucial in DNA damage repair (42, 43). Studies have shown that loss of ARID1A function, such as deletion or mutation (insertion, deletion, nonsense mutation, etc.), affects DNA damage repair (44). SWI/SNF complexes containing ARID1A allow DNA repair proteins to access DNA damage sites (45) efficiently. Inactivation of the SWI/SNF complex by deletion or knockdown of ARID1A impairs DNA DSB repair, increases sensitivity to DNA damaging agents, and impairs γ-H2AX induction (46). In addition, inhibition of ARID1A reduces the recruitment of NHEJ factors (e.g., KU70/KU80 and the ATPase subunit of the SWI/SNF complex) from the DSB locus, thereby affecting the NHEJ process (47). And ARID1A knockout cells were unable to perform effective NHEJ repair after irradiation. It was found that ARID1A is also involved in repairing damaged DNA by homologous recombination (HR). Mechanistically, ARID1A is recruited to the DNA DSB site by interacting with the upstream kinase ATR.ARID1A also helps to recruit the ATPase subunit of the SWI/SNF complex to the DNA damage site. Deletion of ARID1A also impairs the G2/M DNA damage checkpoint (48). In summary, ARID1A promotes DSB end resection and helps maintain checkpoint signaling. Overall, ARID1A protects the genome by interacting with mechanisms of different DNA repair mechanisms.

### ARID1A alterations with PI3K/AKT/mTOR signaling pathway

Activation of the acylinositol triphosphate kinase (PI3K/AKT) signaling pathway is an essential mechanism for the progression of many cancers, and studies have shown that the role of ARID1A in tumors can be associated with activation of the PI3K signaling pathway (49, 50). In ovarian clear cell carcinoma (OCCC), PIK3CA activating mutations occur concurrently with ARID1A deletion mutations (51), and mutations in PIK3CA are present in 46% of ARID1A deletion tumors (52). Mutations in the ARID1A gene in clear cell carcinoma of the ovary activate the PI3K/AKT signaling pathway, which activates the PIK3CA gene and inactivates PTEN, leading to tumorigenesis (51). In addition, the phosphorylation level of AKT was significantly higher in ARID1A-deficient gastric cancer cells than in non-deficient cells, as well as frequent mutations in the PI3K/AKT signaling pathway (deletion of PTEN or activation of PIK3CA) in endometrial carcinomas with deficient ARID1A expression (53). In ARID1A-mutated ovarian cancer cells, the expression of PIK3IP1 was reduced, which led to the activation of the PI3K/AKT/mTOR pathway (50). The above results indicate that ARID1A mutation deletion tends to occur concurrently with PIK3CA gene mutation and activation of the PI3K/AKT signaling pathway, suggesting that small molecule inhibitors of our PI3K/AKT/mTOR signaling pathway could be used as potential targeted therapeutic agents to inhibit ARID1A mutation-deficient tumors.

### ARID1A alteration and mismatch repair, immune checkpoints

ARID1A is involved in DNA mismatch repair, and ARID1A mutations were enriched in MSI-H tumors. It has now been shown that in endometrial and colorectal cancers, ARID1A deficiency is associated with MLH1 silencing due to promoter hypermethylation. It was found that ARID1A interacts with the mismatch repair (MMR) protein MSH2, recruiting MSH2 to chromatin, and when ARID1A undergoes inactivating mutations, it impairs MMR function, leading to increased microsatellite instability and mutations (54). Specifically, ARID1A recruits MSH2 to chromatin during DNA replication and supports mismatch repair (54). ARID1A inactivation attenuates mismatch repair and enhances mutation. Thus, ARID1A deficiency may generate the microsatellite instability genomic signature MSH2 by interacting with perturbations in M. Finally, in homozygous mice, cancers arising from ARID1A-deficient ovarian cancer cell lines exhibited high mutation loads, elevated PD-L1 expression, and increased numbers of lymphocytes in tumor infiltration. Using an anti-PD-L1 antibody significantly reduced tumor burden in ARID1A-deficient mice and prolonged survival in ARID1A wild-type ovarian cancer mice (54).

Although the interaction of ARID1A with MSH2 and the resulting microsatellite instability may be associated with ARID1A-deficient cancer responsiveness to immune checkpoint blockade, other mechanisms may also play a role. Alterations in ARID1A are significantly associated with more prolonged progression-free survival (PFS) after immune checkpoint inhibition in various human cancers, independent of TMB or MSI status (55). Although the mechanistic mechanism by which ARID1A mutations are associated with immune checkpoint blockade benefits is unknown, the interaction of EZH2 methyltransferase with ARID1A may be relevant to the immune response. Indeed, ARID1A interacts with EZH2 and inhibits the EZH2-mediated interferon (IFN) response, which is essential for the immune response (56). It has been shown that alterations in ARID1A are associated with checkpoint gene expression and other markers of immunogenicity in MSS colorectal cancer (57).

These results indicate that ARID1A, as the subunit with the highest mutation rate in the SWI/SNF chromatin remodeling complex family, plays a role in tumor through the regulation of the above biological processes, and has broad application prospects in tumor development and targeted therapy.

### ARID1A alterations and tumor drug resistance

Mutations, down-regulation of expression, and deletion of the ARID1A gene can predict drug resistance and recurrence in various cancers. Chemoresistance in epithelial ovarian cancer patients was associated with reduced expression of ARID1A (58). Bladder cancer patients had P153A mutations in the ARID1A/1B subunit before and after resistance to pazopanib (59). In addition, ARID1A deletion activates ANXA1 protein expression in HER2+ breast cancer cells, which consequently induces resistance to trastuzumab through activation of AKT protein (60). ARID1A knockdown was found to promote the lung adenocarcinoma cell cycle and accelerate cell division (61). Mechanistically, ARID1A knockdown increases the phosphorylation levels of the oncogenic proteins EGFR, ErbB2, and RAF1, inducing bypass activation of the ErbB pathway, activation of the VEGF pathway, and changes in the expression levels of biomarkers of epithelial-mesenchymal transition, leading to disease progression that is insensitive to treatment with epidermal growth factor receptor tyrosine kinase inhibitors (61). ARID1A deletion in ovarian cancer can transcriptionally activate MRP2 protein expression after chromatin remodeling, leading to multidrug resistance to carboplatin and paclitaxel in ovarian cancer (62). Immunohistochemical analysis of ovarian cancer samples also confirmed a strong negative correlation between ARID1A expression and MRP2 expression (62), which provides an opportunity to overcome ARID1A deletion-induced chemoresistance in ovarian cancer by targeting MRP2.ARID1A deletion leads to resistance to PARP inhibitors in ovarian cancer patients (63), significantly increases chemoresistance in squamous cell carcinoma (64), promoting cell proliferation, metastasis, and resistance to sunitinib in clear cell renal cell carcinoma (65).

**Figure 2:**
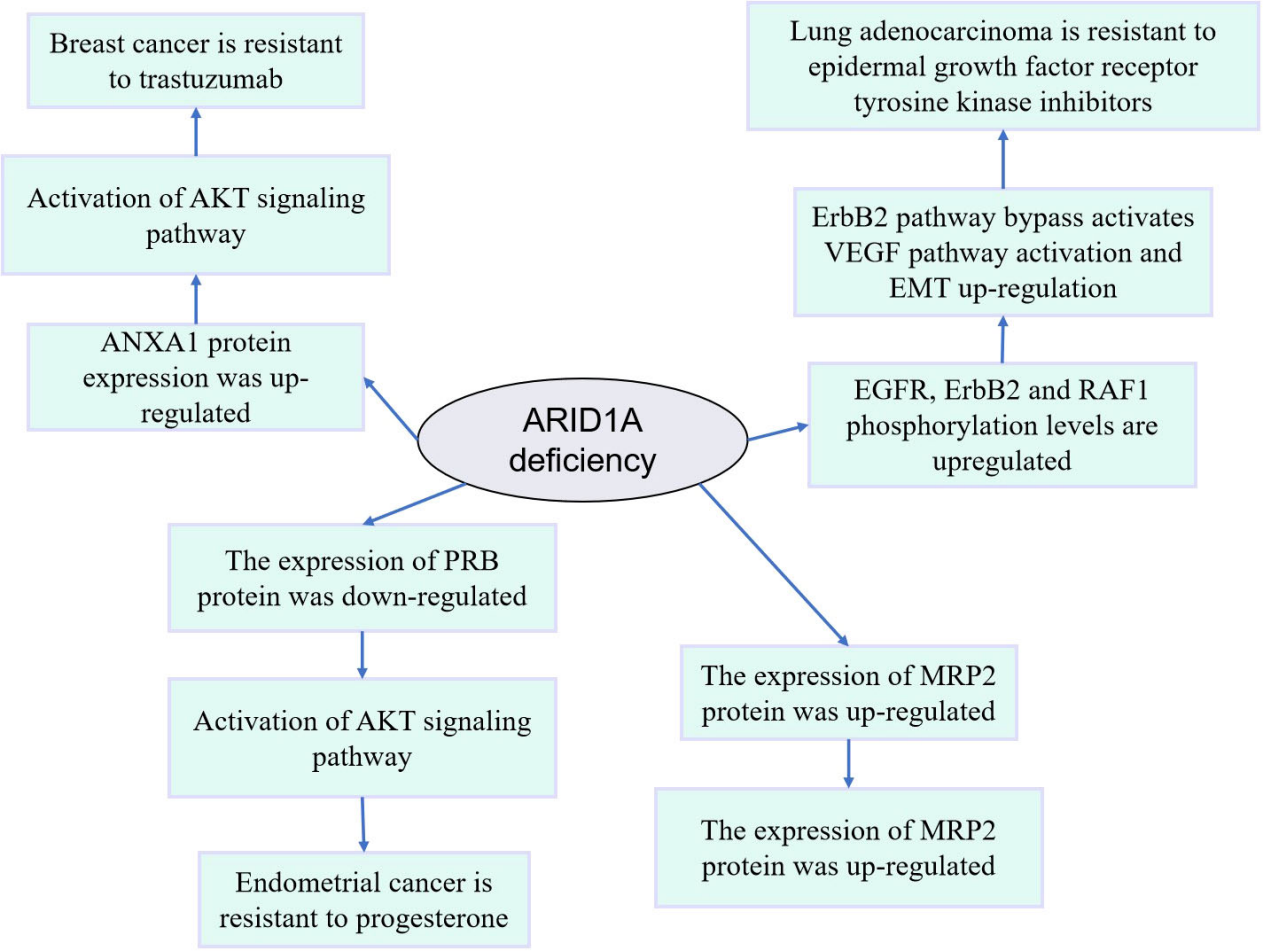
Mechanism diagram of drug resistance induced by abnormal expression or variation of ARID1A.

The above results indicate that the ARID1A-induced tumor drug resistance mechanism study has made significant progress (**Table 1**, **Figure 2**). However, it is still at the protein level without in-depth exploration of other genes and metabolic pathway changes induced by ARID1A alteration. In addition, the above conclusions should be validated in more cancer patients.

**Table 1:** Abnormal expression and variation of ARID1A and SMARCA4 in tumor drug resistance.

Subunit gene	Coding protein	Expression of drug resistance in tumors	Tumor types and drug resistance	References
ARID1A	BAF250A	Mutations Low expression	Cholangiocarcinoma (single nucleotide variant, gemcitabine and cisplatin), bladder cancer (P153A mutation, pazopanib) Ovarian cancer (paclitaxel, carboplatin)	(59, 66) (58)
SMARCA4	BRG1	Deficiency High expression Low expression Deficiency Change the staining, the structure of the mass	Ovarian cancer (PARP inhibitors), squamous cell carcinoma (chemotherapeutics), renal cell carcinoma (Sunitinib), breast cancer (trastuzumab), endometrial cancer (progesterone), ovarian clear cell carcinoma (cisplatin), ovarian cancer (carboplatin and paclitaxel), lung adenocarcinoma (epidermal growth factor receptor tyrosine kinase inhibitors) Non-small cell lung cancer (cisplatin), bone sarcoma cells (doxorubicin) Pullcell lymphoma (eutinib and vennettoc), highly serous ovarian carcinoma (carboplatin) Triple-negative breast cancer (cisplatin), ovarian clear cell carcinoma (EZH 2 inhibitor), ovarian cancer, and lung cancer (cisplatin) Lung adenocarcinoma (oshitinib)	(60–65, 67, 68) (69, 70) (71, 72) (73–75) (76)

## Impact of ARID1A alterations and potential therapeutic targets

**Figure 3:**
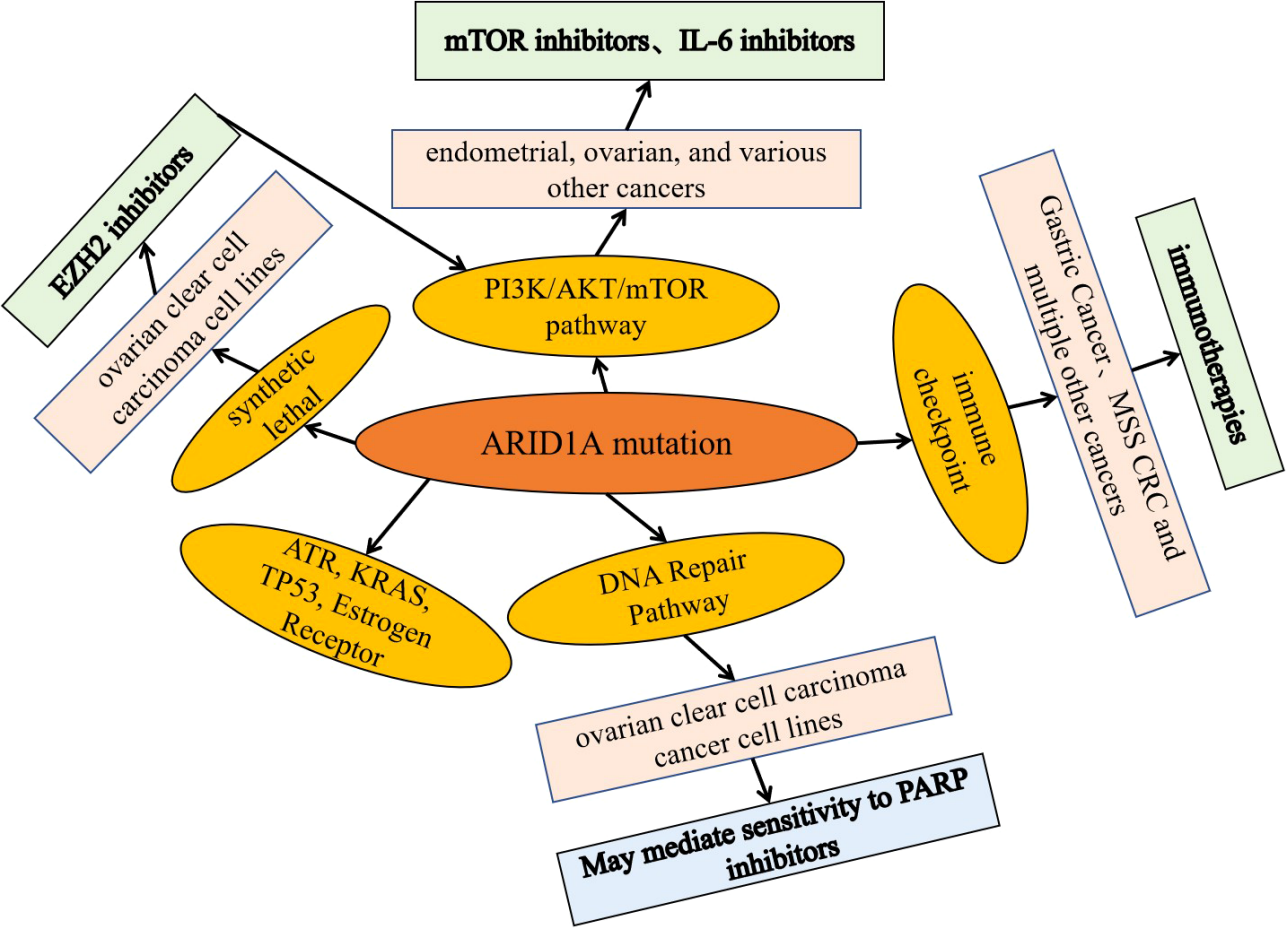
Potential targets of ARID1A mutant tumor phase and basic application of inhibitors. Based on preclinical mechanistic cancer models and/or cancer patient-based data, explore potential therapeutic targets and related inhibitors for tumors targeting different signaling pathways and biological processes, including immune checkpoints, as well as potential therapeutic targets and inhibitors such as mTOR, PARP, ATR, EZH2, and HDACs.

ARID1A, one of the most frequently mutated SWI/SNF subunit genes, is mutated in tumors, leading to loss of protein product function and affecting many signaling pathways and biological processes important in tumorigenesis (24). Currently, based on preclinical mechanistic cancer models and/or cancer patient-based data, potential therapeutic targets for tumors are being explored for different signaling pathways as well as biological processes, including immune checkpoints, as well as potential therapeutic targets such as mTOR, PARP, ATR, EZH2 and HDACs (**Figure 3**).

### ARID1A alterations and potential targets of the PI3K/Akt/mTOR pathway

Data from a preclinical study showed that concurrent ARID1A-PIK3CA mutations in endometrial and ovarian cancers promote ovarian clear cell tumorigenesis through the tumorigenic inflammatory cytokine signaling pathway, which can be blocked by IL-6 inhibitors (77). Whereas mTOR inhibitors such as everolimus inhibit the increased phosphorylation of Akt caused by alterations in ARID1A (50, 78), mechanistically, ARID1A and EZH2 associate with the PIK3IP1 promoter; PIK3IP1 inhibits PI3K/Akt/mTOR signaling.ARID1A, although activating the expression of PIK3IP1, overrides the expression of EZH2, thereby reducing PIK3IP1 expression, and when ARID1A is absent, EZH2 silences PIK3IP1 (50, 78, 79).

### ARID1A alterations sensitize tumors to immune checkpoint blockade

Some clinical data suggest that alterations in ARID1A are found in 73% of EBV-positive gastric cancers and correlate with high PD-L1 expression (80, 81). Data showed that gastric cancers with EBV were more sensitive to immune checkpoint blockade (82). Alterations in ARID1A are associated with a higher tumor mutation burden in various cancers (55, 57, 83), making such cancers or subtypes potentially more susceptible to immunotherapy.

### ARID1A alterations and sensitivity/resistance to PARP inhibitors and platinum resistance

Preclinical data suggest that ARID1A deficiency sensitizes cancer cells to PARP in both *in vitro* and *in vivo* models with inhibitors (47, 48, 54, 84, 85), a process mechanistically linked to DNA damage repair (48, 54). However, the activity of PARP inhibitors in patients with altered ARID1A is unknown. In several breast and ovarian cancer cell lines, ARID1A deletion resulted in resistance to PARP inhibitory therapy (63, 86).

### ARID1A alterations and EZH2 inhibitors, HDAC inhibitors

Some preclinical data suggest that inhibition of EZH2 may be effective in ovarian cancer, and mechanistically, inhibition of EZH2 histone methyltransferase activity is synthetically lethal in ARID1A mutant cancers (87, 88). Some preclinical data suggest that HDAC inhibitors, such as SAHA, inhibiting ARID1A mutation-deficient tumors may be effective. Mechanistically, ARID1A mutations are sensitive to pan-HDAC inhibitors such as SAHA. This is associated with enhanced growth inhibition induced by inhibition of HDAC2 activity in ARID1A mutant cells. HDAC2 interacts with EZH2 in an ARID1A state-dependent manner (89–91).

### ARID1A alterations and other targets: ATR, KRAS, TP53, Estrogen Receptor

Preclinical data suggest a synthetic lethal interaction between targeted ATR therapy and ARID1A deletion mutations in ovarian clear cell carcinoma and other cancer cell lines (84, 92–94). It was found that knockdown of ARID1A containing remodeling complexes may be active in kras mutant colorectal cancer, mechanistically due to the involvement of ARID1A in K-RAS downstream (nuclear) signaling (95). Furthermore, some exclusivity between wild-type TP53 and ARID1A mutations suggests that restoration of wild-type p53 could benefit a combinatorial therapeutic approach (96). Similarly, in ER-positive breast cancer, loss of ARID1A determines resistance to the ER-degrading agent fulvestrant (97).

Taken together, high-frequency mutations of ARID1A in tumors lead to loss of function of the protein product and affect many necessary signals in tumorigenesis. For example, the PI3K/Akt/mTOR pathway, the KRAS pathway, DNA damage repair, EZH2, and immune function (including interactions with the MSH2 mismatch repair gene product). Compounds targeting these pathways may be active based on preclinical mechanistic cancer models and/or based on cancer patient data, including immune checkpoint blockade (anti-PD-1/PD-L1) and inhibitors of mTOR, PARP, ATR, EZH2, and HDAC (47, 48, 54, 84, 85). Studying the function of ARID1A is essential for a more precise understanding of the function of ARID1A, as well as matching cancer patients carrying ARID1A mutations with homologous drugs to optimize efficacy.

In conclusion, ARID1A is an essential component of the SWI/SNF chromatin remodeling complex and a tumor suppressor mutated or absent in many tumors. However, the specific oncogenic mechanism of ARID1A is still unclear, and the variability of ARID1A mutations in different tumors and the impact of ARID1A mutations on tumor prognosis need to be investigated in depth. An in-depth study of the function of the ARID1A gene and its specific molecular mechanism of action will help to increase the understanding of the biological role of chromatin remodeling complexes and provide new ideas for cancer diagnosis and treatment.

## Structure of SMARCA4 and its function in tumors

The SMARCA4 gene is located on chromosome 19p13.2 and encodes a transcriptional activation protein, which is also known as BRG1 (98). The SMARCA4 protein has a bromodomain and a deconjugating enzyme/ATPase. The bromodomain is a protein structural domain of approximately 110 amino acids recognizing acetylated lysine residues, such as those found at the N-terminal end of histones; these structural domains have a role in the regulation of gene transcription (99). Deconjugases are enzymes that bind and can remodel nucleic acids or nucleic acid-protein complexes. ATPase is an enzyme that catalyzes the hydrolysis of the phosphate bond in adenosine triphosphate (ATP) to form adenosine diphosphate (ADP); they utilize the energy released from the decomposition of the phosphorylated bond and use it to carry out other cellular reactions (100).

SMARCA4 (BRG1) forms a subunit in several different proteomes known as the SWI/SNF protein complex. The SWI/SNF complex regulates gene activity/expression through the process of chromatin remodeling. Chromatin is an arrangement of proteins and DNA that packages DNA into chromosomes. The chromatin structure can be remodeled to adjust how tightly DNA is wrapped. Chromatin remodeling is one of the key ways to control gene expression during development. Tightly packed DNA represses gene expression compared to loosely packed DNA.SMARCA4 utilizes ATP to provide energy for chromatin remodeling (99, 101).

SMARCA4 is involved in various biological normal and tumor tissue processes through its ability to regulate gene activity. It has diverse functions, including transcriptional regulation, DNA damage repair, differentiation, and mitosis. Thus, it plays an essential role in the cells of regenerating organisms. SMARCA4 was found to function with BRM and interact with the protein product of the retinoblastoma tumor suppressor gene to inhibit the function of the E2F transcription factor (102). Although SMARCA4 is a tumor suppressor, it has been shown that SMARCA4 expression is upregulated in cancer compared to healthy tissues. Thus, SMARCA4’s role as a tumor suppressor is not its only role in cancer, and SMARCA4 has also been found to be an oncogene (103). Indeed, SMARCA4 overexpression in cancer can serve as a prognostic indicator. In most cancers, including breast, colorectal, and prostate cancers, SMARCA4 upregulation is associated with poorer prognosis, suggesting that SMARCA4 is an oncogene (101, 103). However, patients with non-small cell lung cancer (NSCLC) caused by SMARCA4 deletion have a worse prognosis than those with normal SMARCA4 expression (104). Thus, SMARCA4 has different roles in different cancer types.

### SMARCA4 acts in tumors through autophagy and apoptosis pathways

A link between SMARCA4 and autophagy proteins was found, with autophagy significantly reduced in cells lacking SMARCA4 expression (105, 106). Furthermore, SMARCA4 deficiency was directly associated with reduced critical autophagy-regulated genes, mainly ATG16L1, ATG7, AMBRA1, and WIPI2.ATG16L1 and ATG7 are direct components of the ATG16L1-ATG5-ATG12 coupling system, and these proteins contribute to LC3 phosphatidylethanolamine coupling and autophagy cell formation (105, 106).Ambra1 stabilizes the VPS34/Beclin1 complex by binding to Beclin1 and aids in the maturation of autophagic vesicles. It has been hypothesized that AMBRA1 is required for autophagy and that AMBRA1 deficiency correlates with suppression of autophagic flux (105, 106).WIPI2 is one of four WIPI proteins that make up the support proteins. WIPI acts as an autophagic signaling modality that binds to PI3P.WIPI aids in LC3 through the recruitment and binding of ATG16L1-ATG5-ATG12 complexes lipolysis to aid in the lipidation of LC3 (107). Studies have shown that SMARCA4 and p53 can interact (108, 109). p53 is a regulatory protein involved in autophagy and apoptosis (110, 111). It has been found that in colorectal cancer, SMARCA4 binds to SIRT-1 and enhances SIRT-1-mediated deacetylation of p53 at the K382 locus. Knockdown of SMARCA4 reduces the efficiency of SIRT-1 deacetylation and increases p53 stability (108). Another study showed that in CRC, SMARCA4 knockdown resulted in increased p53 expression and found that the SMARCA4/CHD4/HDAC1 complex regulates p53 transcription and stability (109). Retinoblastoma protein (RB) was found to interact with SMARCA4 and inhibit autophagy. RB is an oncogenic protein that induces cell cycle block at the G1 checkpoint, and RB together with SMARCA4 inhibits E2F1 activation (100, 112).SMARCA4/R B inhibits E2F1 to initiate autophagy. In addition, SMARCA4 has been found to enhance the inhibitory effect of RB on E2F and cyclinA. This suggests that SMARCA4 may further interact with RB to initiate autophagy. However, it has also been found that overexpression of SMARCA4 inhibits autophagy and that the inhibitory process is achieved through activation of the WNT/β-cyclin signaling pathway. This suggests the need to investigate further the role of this pathway in autophagy in the context of SMARCA4 and autophagy SMARCA4 in different cancer types. In colorectal cancer, inhibition of SMARCA4 led to activation of the JNK pathway (113). Furthermore, in a study of melanoma cells exposed to UV light, SMARCA4 and MITF inhibited apoptosis and enhanced the trans-responsive effects of the melanoma apoptosis inhibitor ML-IAP (114). In addition, a reduction in SMARCA4-related target genes, including the protein KLK2, a known prostate cancer apoptosis inhibitor, was found in SMARCA4-deficient prostate cancer cells (115). Furthermore, in pancreatic ductal adenocarcinomas (PDAs) with intraepithelial neoplasia (panIN) originating from KRAS mutant cells, SMARCA4 was found to promote the initiation of panIN and its progression to pancreatic cancer (116). BRG1 inhibition has been observed to prevent panIN formation and block panIN-derived PDAs by inducing apoptosis.SMARCA4 binds to the SOX9 promoter and helps to initiate the transcription of SOX9.SOX9 has been shown to affect tumor proliferation, and SOX9 knockdown has a pro-apoptotic effect (117). Although the mechanism by which BRG1 inhibits apoptosis has not been further investigated, SOX9 may be another factor that inhibits apoptosis through the Wnt/β-linker protein signaling pathway (117).

The above study suggests that SMARCA4 is related to autophagy and affects apoptosis. It is possible that SMARCA4 promotes or inhibits tumor cell growth. However, SMARCA4 has different roles in different stages of autophagy. More research on the mechanism of SMARCA4’s role in different cancer types is needed in future studies.

### SMARCA4 alterations and tumor drug resistance

Studies have shown that SMARCA4 deletion or low expression can induce drug resistance in some tumors. Currently, clinically significant resistance to the combination of ibrutinib and vinblastine is observed in patients with mantle cell lymphoma (MCL). Mechanistic studies have found that knockdown of SMARCA4 in MCL cells leads to deletion of the transcriptional repressor of the BCL2L1 gene, which transcriptionally upregulates the BCL2L1 gene and increases the BCL2L1 protein, resulting in significant resistance to the combination of ibrutinib and vinotec in MCL cells (71). In addition, it was found that the knockdown of SMARCA4 in triple-negative breast cancer (TNBC) resulted in hyperactivation of YAP1 and upregulation of EMT (73). This suggests that SAMRCA4 mediates EMT-induced cisplatin resistance by activating YAP1 in TNBC.

In addition to the separate study of SMARCA4 described above, studies have shown that high-grade serous carcinoma (HGSC) cells with a low SMARCA4/high SMARCA2 expression phenotype are highly resistant to carboplatin. Analysis of the mechanism showed that altered chromatin accessibility in HGSC cells resulted in activation of the FGFR1- pERK1/2 signaling pathway and overexpression of the anti-apoptotic gene BCL2, causing chemoresistance (72). In addition, SMARCA4/2 deletion reduces the expression of the Ca2+ channel IP3R3, which impairs the transfer of Ca2+ from the endoplasmic reticulum to the mitochondria required to induce apoptosis, leading to cisplatin resistance in ovarian and lung cancer (74). It was shown that switching SMARCA4 to SMARCA2 induced acquired resistance to EZH2 inhibitors in ARID1A-mutated ovarian clear cell carcinoma. In cell death/apoptosis signaling, the anti-apoptotic gene BCL2 is a direct target of SMARCA4, and SMARCA4 deletion upregulates the expression of the anti-apoptotic gene BCL2 in EZH2 inhibitor-resistant cells, resulting in the upregulation of BCL2 mRNA and protein levels. Thus, SMARCA4 deletion is associated with reduced death/apoptosis features in drug-resistant cells (75). Interestingly, the ATPase activity of the SMARCA4/SMARCA2 subunit can also promote resistance to oxitinib treatment in EGFR-mutated lung adenocarcinomas by altering chromatin openness (76).

**Figure 4:**
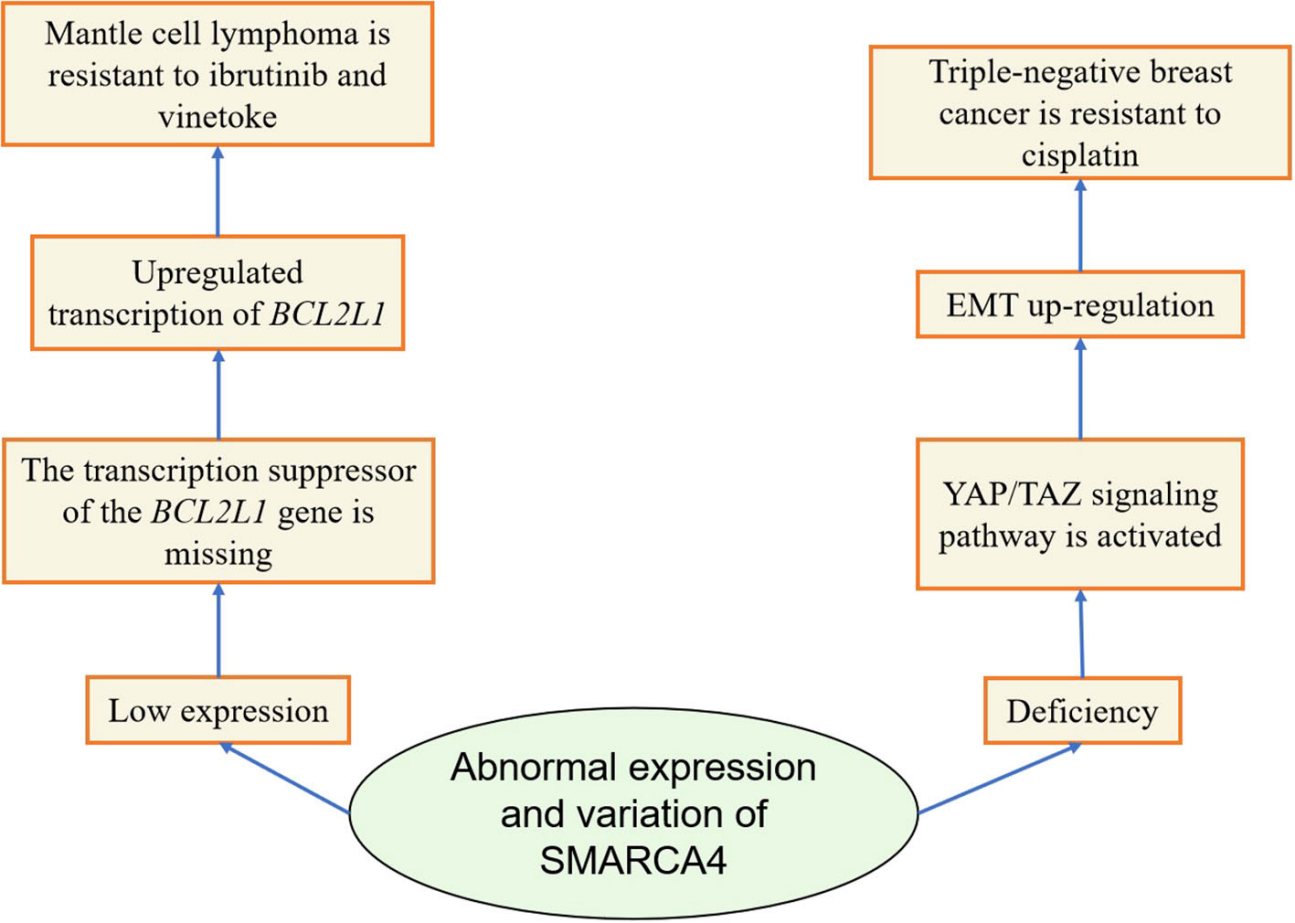
Diagram of the mechanism by which abnormal expression or variation of SMARCA4 induces tumor resistance.

The above studies systematically elucidated the changes of SMARCA4 in drug resistance in various tumors (**Table 1**, **Figure 4**). However, they did not delve into why the changes of the SMARCA4 gene are different in different cancer types, whether it is just due to the differences in tumor types or whether all the various abnormalities of SMARCA4 induce drug resistance in tumors through different mechanisms, which needs to be explored further.

## Impact of SMARCA4 alterations and potential therapeutic targets

Alterations in SMARCA4 result in an abnormal SWI/SNF complex, an abnormal chromatin remodeling complex that can affect the transcription of interferon-stimulated genes critical for immune responses and the differentiation, activation, and recruitment of several immune cell types. In addition, various other signals have been investigated for potential pharmacological intervention (118).

### SMARCA4 alterations and immunotherapy with ICIs

Immunotherapy with ICIs has emerged as a promising treatment modality for SMARCA4-altered cancers. Studies have reported that ICI treatment is associated with a significantly improved prognosis in SMARCA4-abnormalized non-small cell lung cancer (119). Some ICI responses were seen in patients with high tumor mutation load (TMB) and/or high PD-L1 expression by IHC, both of which are markers of response to immunotherapy, but other patients with low TMB and PD-L1 negativity still responded (120–124).

### SMARCA4 alterations and EZH2 inhibitors, HDAC inhibitors

Studies have shown that SWI/SNF deficiency leads to elevated PRC2, a potential therapeutic target for SMARCA4 abnormalities. Indeed, SMARCA4-deficient cancer cells showed sensitivity to inhibition of methyltransferase EZH2, the catalytic subunit of PRC2. In SCCOHT cells, EZH2 inhibitors effectively suppressed the growth of xenografts in SCCOHT cell lines (125). Targeting histone modification complexes in SCCOHT patients has also shown promising therapeutic promise. Re-expression of SMARCA2 due to HDAC inhibitors in SCCOHT inhibits SCCOHT cell proliferation, including in an *in vivo* xenograft model of SCCOHT cells, which respond to the HDAC inhibitor quintet (126).

### SMARCA4 alterations and cell cycle protein inhibitors

SCCOHT cells are also sensitive to cell cycle protein-dependent kinase 4/6 (CDK4/6) inhibition. Deletion of SMARCA4 leads to down-regulation of cell cyclin D1, which restricts CDK4/6 kinase activity in SCCOHT cells and leads to sensitization to CDK4/6 inhibitors *in vitro* and *in vivo* (127). A similar synthetic lethal interaction between SMARCA4 deficiency and CDK4/6 inhibition was identified in SMARCA4-deficient NSCLC (128).

### SMARCA4 alterations and DNA damage repair inhibitor

SMARCA4 binds to BRCA1, an essential gene product for DNA damage repair (129). Furthermore, preclinical data suggest that SMARCA4-deficient lung cancer cells exhibit enhanced replication stress. Exposure to p-ATR inhibitors (impairing DNA repair) would lead to replication catastrophe in these cells (130). Similarly, low expression of SMARCA4 significantly correlates with platinum-based chemoselectivity. The rapid reactivity of NSCLC may be due to platinum-induced extensive DNA damage (69).

In conclusion, targeted therapy using EZH2, HDAC, CDK4/6 inhibitors, DNA damage repair inhibitors, and immunotherapy with ICIs is mechanistically reliable and has some application prospects.

## Advances in tumor targeting drugs targeting alterations in SMARCA4 subunits

A genome-wide CRISPR screen in SMARCA4 mutant lung cancer cell lines demonstrated that loss of MCL1 can sensitize SMARCA4 mutant lung cancer cells to SMARCA2 degradation and that the SMARCA2 degrader PRT3789 coupled with the MCL1 inhibitor PRT1419 synergistically interacts with and does not affect SMARCA4 mutant lung cancer models. SMARCA4 wild-type cells. PRT3789 is highly selective for SMARCA2 (over SMARCA420-fold) and tolerated well *in vivo* and *in vivo* models (131, 132).

FHD-286 is an oral inhibitor of the BAF complex for treating AML and uveal melanoma (UM).FHD-286 inhibits BAF function by inhibiting the ATPase components of the BAF complex, SMARCA4, and SMARCA2 (133, 134). Inhibition of SMARCA2/SMARCA4 by FHD-286 affects the level of SPI1 transcription and its downstream transcriptional program, which in turn affects cell proliferation and cell survival. SPI1 is an ETS family of transcription factors that play a crucial role in hematopoietic development and differentiation, and modulation of SPI1 expression has been implicated in the tumourigenesis of acute myeloid leukemia (AML) (133, 134). FHD 286 responds well in AML patients, with cell killing comparable to standard therapy and dose-dependent inhibition of tumor growth (133, 134). FHD-286 is currently in Phase I clinical trials for AML/MDS. In 85% of uveal melanomas (UM) with GNAQ/GNA11 mutations, which overexpress the transcription factors MITF and SOX10 and over-interact with the BAF complex, FHD-286 inhibits SMARCA2/SMARCA4, leading to loss of accessibility of the SOX10 and MITF transcription factor binding sites, and suppression of the SOX10 and MITF dependent gene (GNAQ/GNA11) expression and preclinical data showed that FHD-286 dose-dependently inhibited tumor growth (133, 134).

Components of the SWI/SNF complex are mutated in some cancers but rarely in prostate cancer. Prostate cancers driven by androgen receptor (AR) or FOXA1 are more sensitive to SWI/SNF degraders than cancers in which subunits of the SWI/SNF complex are mutated (135). AU-15330 is a protein hydrolysis-targeted chimeric degrader of the ATPase subunits of the SWI/SNF complex (SMARCA2 and SMARCA4). The main mechanism of action is degradation of SMARCA2 and SMARCA4, blocking access to chromatin, compression of chromatin around the core enhancers of AR, FOXA1, ERG, and MYC, and transcription factors are prevented from binding to cancer-driving enhancers, thereby attenuating the pro-oncogenic factor transcriptional program (135).AU-15330 is effective in prostate cancer xenograft models in inhibiting tumor growth and synergizes with the AR antagonist enzalutamide (135).

ACBI-1 is a PROTAC technology-based BAF ATPase subunit SMARCA2 and SMARCA4 degradant, is also a PBAF member PBRM1 degradant, and can effectively inhibit the proliferation of tumor cells (136). SMARCA4 has been found to be a unique factor in medium to long-term (but not short-term) tumor cell survival in alveolar rhabdomyosarcoma (ARMS), and ACBI-1 shows similar long-term tumor cell dependence *in vitro* and *in vivo* (137).

In summary, the above inhibitor-targeted therapies are mechanistically sound as they show better biological activity in basic *in vitro* models (**Table 2**). However, for cancer patients with malignant tumors with SMARCA4 alterations, more downstream targets need to be demonstrated, which have been used in conjunction with their clinical coadministration, an approach that has a more long-term therapeutic outlook for the treatment of cancer.

**Table 2:** SMARCA4 subunit mutation tumor inhibitor development.

Target site	Small Molecule Inhibitor Name	Comment	References
SMARCA bromodomain	PFI-3	The inhibitor did not exhibit effective antiproliferative activity.	(138)
ATPase structural domain	FHD-286	The inhibitor had good antitumor activity and was well tolerated in xenograft tumor models.	(133, 134)
SMARCA-PROTAC	ACBI-1	SMARCA2/4 dual-targeting PROTAC degraders that effectively inhibit tumor cell proliferation.	(136, 137)
SMARCA-PROTAC	ACBI-2	Able to effectively inhibit tumor cell proliferation with clear selectivity compared to ACBI1.	(139)
SMARCA-PROTAC	PRT3789	First PROTAC-degrading agent targeting SMARCA2 degradation to enter clinical trials achieves antiproliferative activity against SMARCA4-deficient tumors *in vitro* and *in vivo*.	(131, 132)
SMARCA-PROTAC	AU-15330	AU-15330 effectively inhibits tumor growth and synergizes with the AR antagonist enzalutamide in a prostate cancer xenograft model.	(135)
SMARCA-PROTAC	SMD-3040	With SMARCA4-deficient tumor cell selectivity, it can effectively inhibit the growth of SAMRCA4-deficient tumors and is well tolerated.	(140)
SMARCA-PROTAC	A947	Effectively inhibited the growth of SAMRCA4 mutant tumors but showed little growth inhibition of SMARAC4 wild-type tumors.	(141)

## Discussion

The SWI/SNF complex has a complex structure in organisms that affects various physiological activities of cells, plays a vital role in various life activities of cells, and its subunit abnormalities are involved in developing many malignant tumors. Currently, studies on the mechanism of SWI/SNF hypermutated subunits ARID1A and SMARCA4 mainly focus on their regulation of gene expression, but whether it is involved in other cellular activities is unclear. Moreover, the bias of ARID1A and SMARCA4 in different tumors and their pro- or anti-cancer effects in different tumors and different processes of tumor development have led to the incomplete study of ARID1A and SMARCA4 at present.

Epigenetic regulation is considered a promising target for the treatment of human cancers. Chromatin remodeling is one of the epigenetic modalities, and chromatin remodeling proteins play essential roles in mobilizing nucleosomes, regulating transcription, DNA replication, and repair. Among them, the SWI/SNF chromatin remodeling complex has the highest frequency of mutations in human cancers. Clinical treatments for ARID1A and SMARCA4 mutant tumors, as well as small molecule inhibitors, are available, but there are still some basic research and clinical treatment-related questions that need to be explored, e.g., can ARID1A mutant breast and ovarian cancer cell lines and high-grade plasmacytoid ovarian cancers, which have a certain degree of resistance to PARP inhibitors, be used in combination with a certain drug to increase their sensitivity? Gemcitabine is effective in ARID1A-mutated ovarian clear cell carcinoma cell lines and OCCC patients. However, the mechanism of action is unknown, and analyzing its mechanism will help us to achieve precision therapy better.

In conclusion, the highly mutated subunits of the SWI/SNF complex, ARID1A and SMARCA4, have essential roles in tumorigenesis and development. An in-depth study of the role of SWI/SNF complex high mutation subunits ARID1A and SMARCA4 in tumors and their mechanisms is of great significance, providing new directions for early diagnosis or targeted therapy of tumors by clinical ARID1A and SMARCA4 mutations.
